# Identification of paternal uniparental disomy on chromosome 22 and a *de novo* deletion on chromosome 18 in individuals with orofacial clefts

**DOI:** 10.1002/mgg3.459

**Published:** 2018-08-23

**Authors:** Ganiyu O. Oseni, Deepti Jain, Peter A. Mossey, Tamara D. Busch, Lord J.J. Gowans, Mekonen A. Eshete, Wasiu L. Adeyemo, Cecelia A. Laurie, Cathy C. Laurie, Arwa Owais, Peter B. Olaitan, Babatunde S. Aregbesola, Fadekemi O. Oginni, Saidu A. Bello, Peter Donkor, Rosemary Audu, Chika Onwuamah, Solomon Obiri‐Yeboah, Gyikua Plange‐Rhule, Olugbenga M. Ogunlewe, Olutayo James, Taiye Halilu, Firke Abate, Lukman O. Abdur‐Rahman, Abimbola V. Oladugba, Mary L. Marazita, Jeffrey C. Murray, Adebowale A. Adeyemo, Azeez Butali

**Affiliations:** ^1^ Department of Plastic Surgery Ladoke Akintola University of Science and Technology Osogbo Nigeria; ^2^ Department of Biostatistics Genetic Coordinating Center University of Washington Seattle Washington; ^3^ Department of Orthodontics University of Dundee Dundee UK; ^4^ Department of Pediatrics University of Iowa Iowa City Iowa; ^5^ Kwame Nkrumah University of Science and Technology Kumasi Ghana; ^6^ School of Public Health Addis Ababa University Addis Ababa Ethiopia; ^7^ Department of Oral and Maxillofacial Surgery University of Lagos Lagos Nigeria; ^8^ Department of Pediatric Dentistry University of Iowa Iowa City Iowa; ^9^ Department of Oral and Maxillofacial Surgery Obafemi Awolowo University Ile Ife Nigeria; ^10^ State House Clinic Abuja Nigeria; ^11^ Department of Virology Nigerian Institute of Medical Research Lagos Nigeria; ^12^ Division of Pediatric Surgery Department of Surgery University of Ilorin Ilorin Nigeria; ^13^ Department of Biostatistics University of Nigeria Nsukka Nigeria; ^14^ Department of Oral Biology Center for Craniofacial and Dental Genetics University of Pittsburgh Pittsburgh Pennsylvania; ^15^ Department of Human Genetics University of Pittsburgh Pittsburgh Pennsylvania; ^16^ National Human Genomic Research Institute Bethesda Maryland; ^17^ Department of Oral Pathology, Radiology and Medicine University of Iowa Iowa City Iowa

**Keywords:** cleft lip and palate, deletions, GWAS, uniparental disomy

## Abstract

**Background:**

Orofacial clefts are the most common malformations of the head and neck region. Genetic and environmental factors have been implicated in the etiology of these traits.

**Methods:**

We recently conducted genotyping of individuals from the African population using the multiethnic genotyping array (MEGA) to identify common genetic variation associated with nonsyndromic orofacial clefts. The data cleaning of this dataset allowed for screening of annotated sex versus genetic sex, confirmation of identify by descent and identification of large chromosomal anomalies.

**Results:**

We identified the first reported orofacial cleft case associated with paternal uniparental disomy (patUPD) on chromosome 22. We also identified a *de novo* deletion on chromosome 18. In addition to chromosomal anomalies, we identified cases with molecular karyotypes suggesting Klinefelter syndrome, Turner syndrome and Triple X syndrome.

**Conclusion:**

Observations from our study support the need for genetic testing when clinically indicated in order to exclude chromosomal anomalies associated with clefting. The identification of these chromosomal anomalies and sex aneuploidies is important in genetic counseling for families that are at risk. Clinicians should share any identified genetic findings and place them in context for the families during routine clinical visits and evaluations.

## 1 INTRODUCTION

Orofacial clefts can be classified as syndromic and nonsyndromic clefts. Syndromic clefts (SC) are clefts with other structural and cognitive phenotypes and they account for 30% of all clefts. There are over 500 Mendelian clefting syndromes currently indexed in OMIM (www.omim.org), with other causes secondary to environmental teratogens, chromosomal anomalies or sporadic events of unknown etiology.

Nonsyndromic clefts (NSC) are the most common forms of clefts accounting for 70% of all clefts (Marazita et al., 2002). NSC affect 1/700 live births worldwide and prevalence varies significantly due to ethnicity and geographical locations. The etiology of NSC is complex and many genes have been reported to be associated (Dixon, Marazita, Beaty, & Murray, 2011). In addition to genes, environmental factors such as smoking have been identified as teratogens that increase the risk for clefting (Little, Cardy, & Munger, 2004).

Chromosomal abnormalities have been reported in both isolated clefts and clefts with associated congenital anomalies. In fact, almost all clefts with associated congenital anomalies have chromosomal abnormalities (Maarse et al., 2012). The prevalence of clefts with associated anomalies and chromosomal abnormalities varies by cleft types and time of diagnosis (prenatal and postnatal). For instance, reported prenatal rates for cleft lip (CL) are 33.3%, cleft lip and palate (CLP) is 50–63.3 and CP is 100%. Postnatal rates for CL are 10.4–22.2%, CLP is 5–31%, and CP is 14%–18%. postnatally (Calzolari et al., 2007; Kallen, Harris, & Robert, 1996; Rittler et al., 2011; Tan et al., 2009; Walker, Ball, Babcook, & Feldkamp, 2001). Furthermore, reported prenatal rates for isolated clefts with chromosomal abnormalities are 5.3%–7.1% for CLP (Maarse et al., 2012; Nyberg, Sickler, Hegge, Kramer, & Kropp, 1995) and postnatal rates are 1.8% for CL, and 1% for CLP (Rittler et al., 2008; Rittler et al., 2011).

Paternal Uniparental Disomy (patUPD) is a situation whereby an individual has inherited a pair of homologous chromosomes from the father (Engel, 1980). patUPD can arise through multiple mechanisms: (a) Trisomy rescue (TR), when a trisomic zygote forms from a disomic sperm with two paternal chromosomes and a normal ovum, followed by subsequent loss of the maternal chromosome; (b) Gamete complementation (GC),where a disomic sperm fertilizes a nullisomic egg missing a chromosome, resulting in a normal chromosome count; (c) Monosomy rescue (MR), when a monosomic sperm fertilizes a nullisomic egg producing a monosomic zygote followed by duplication of the paternal chromosome, (d) Postfertilization mitotic nondisjunction (Mit), leading to mosaicism for trisomic and monosomic cell lines with subsequent duplication in the monosomic line (Liehr, 2014).

Although paternal and maternal uniparental disomy (UPD) in orofacial clefts are very rare, they have been reported on chromosomes 6, 7,10, 12,15 16 and 21 (Hahnemann, Nir, Friberg, Engel, & Bugge, 2005; Kotzot, 2004; Kotzot, 2008; Leslie et al., 2015; Romanelli et al., 2011; Salahshourifar et al., 2010; Tsai, Gibby, Beischel, McGavran, & Johnson, 2004). However, none have been reported on chromosome 22.

To identify variation associated with presumed nonsyndromic clefts in a sub‐Saharan African population, we genotyped samples from affected cases, case family members and unrelated controls. We hypothesized that chromosomal abnormalities are present in some individuals with nonsyndromic clefts and that these individuals will need to be excluded before genome‐wide association studies for clefting are conducted. In our preliminary analyses and data quality control (QC) process, we identified individuals with chromosomal anomalies on several chromosomes including uniparental disomy on chromosome 22, large chromosomal deletions and duplications.

## 2 METHODS

### 2.1 Ethical approval and sample collection

Eligible subjects are individuals who have nonsyndromic OFC and were born to Ghanaian, Ethiopian, and Nigerian parents. The offspring of Caucasians and Asians were excluded. Eligible cases were identified following IRB approval through the free clefts surgical repair projects. Currently, the network for treatment of clefts in Africa is enhanced, due to the efforts of the Pan African Association for Cleft Lip and Palate (PAACLIP). This network is supported by cleft charities, and all members use a common, standardized protocol for phenotyping. In addition, the grant requires that surgeons at all centers perform standardized physical examinations and take clinical photographs, and that full descriptions of the cleft phenotypes and all other recognizable malformations are entered into a clinical database. Signed informed consent were obtained from all participating families, and every family recruited into the study was assigned a unique identifier number (UNID). Data from all recruited families were entered remotely at each center in Africa, into a secured REDcap database (REDCap) database (Harris, Taylor, Thielke, Gonzalez, & Conde, 2009). Deidentified (except for the UNID) samples were shipped from sites in Africa to the United States. Ethical approval was obtained from the institutional review boards at the Lagos University Teaching Hospital Idi‐Araba, Lagos (IRB approval number:ADM/DCST/HREC/VOL.XV/321), Obafemi Awolowo University Teaching Hospital Ile‐Ife (IRB approval number: ERC/2011/12/01), Kwame Nkrumah University of Science and Technology (IRB approval number: CHRPE/RC/018/13), and Addis Ababa University (IRB approval number: 003/10/surg). Samples were XY‐genotyped as part of the quality control system, and DNA concentration is measured using the Qubit (Thermo Fisher Scientific, Grand Island, New York, USA).

### 2.2 DNA extraction and preliminary quality control

Saliva samples were labeled at the Butali laboratory in Iowa and assigned a unique identification (UNID) number prior to DNA extraction. DNA extraction was done at the Butali lab using the Murray lab protocol (genetics@uiowa.edu). Each sample was quantified using Qubit (http://www.invitrogen.com/site/us/en/home/brands/Product-Brand/Qubit.html) and divided into a stock and several working aliquots. This set‐up allowed us to verify sample identity using the stock if mislabeling or cross‐contamination of the working aliquot was suspected during sample handling. As a preliminary quality check, gender reported in the Redcap database was confirmed using Taqman XY genotyping. A 25ul aliquot of consented samples with confirmed genetic sex and DNA concentration of ≥ 50ng/ul was sent for MEGA array genotyping at the Center for Inherited Disease Research (CIDR).

### 2.3 Genotyping

The expanded Illumina Multi‐Ethnic Genotyping Array (MEGA) v2 15070954 A2 (genome build 37) that contains over 2 million Single Nucleotide Polymorphisms and over 60,000 rare variants selected from populations of African origin was used for genotyping. Genotyping was carried out on 3,347 samples which included 3,198 unique samples and 70 duplicates. HapMap controls (70 unique samples and 9 duplicates) were also genotyped as part of the quality control process.

### 2.4 Data cleaning

The goal of the data cleaning process was to identify a high‐quality genotype dataset that can be used for detecting significant genotype associations with nonsyndromic clefts. This process included sex chromosome checks, a check for missing call rates, batch effects, identification of large chromosomal anomalies, confirmation of relatedness (i.e., identity by descent) and establishment of continental ancestry with respect to HapMap samples using methods described in Laurie et al. (2010) and implemented using R packages GWAS Tools (Gogarten et al., 2012), SNPRelate (Zheng et al., 2012) and GENESIS (Conomos & Thornton, 2016). Large chromosomal anomalies, such as aneuploidy, copy number variations and mosaic uniparental disomy, can be detected using “Log R Ratio” (LRR) and “B Allele Frequency” (BAF) (Conlin et al., 2010; Peiffer et al., 2006). LRR is a measure of relative signal intensity (log2 of the ratio of observed to expected intensity, where the expectation is based on other samples). BAF is an estimate of the frequency of the B allele of a given single nucleotide polymorphism (SNP) in the population of cells from which the DNA was extracted. In a normal cell, the B allele frequency at any locus is either 0 (AA), 0.5 (AB) or 1 (BB) and the expected LRR is 0. Both copy number changes and copy‐neutral changes from biparental to uniparental disomy (UPD) result in changes in BAF, while copy number changes also affect LRR.

To identify aneuploid or mosaic samples systematically, we used the “Circular Binary Segmentation” (CBS) (Venkatraman & Olshen, 2007) and identification of runs of homozygosity. For anomalies that split the intermediate BAF band into two components, we used CBS on BAF values for SNPs not called as homozygotes. For heterozygous deletions (with loss of the intermediate BAF band), we identified runs of homozygosity accompanied by a decrease in LRR (Laurie et al., 2012). All sample‐chromosome combinations with anomalies greater than 5 Mb or sample‐chromosome combinations with the sum of the lengths of the anomalies greater than 10 Mb were verified by manual review of BAF and LRR plots.

## 3 RESULTS

**Figure 1:**
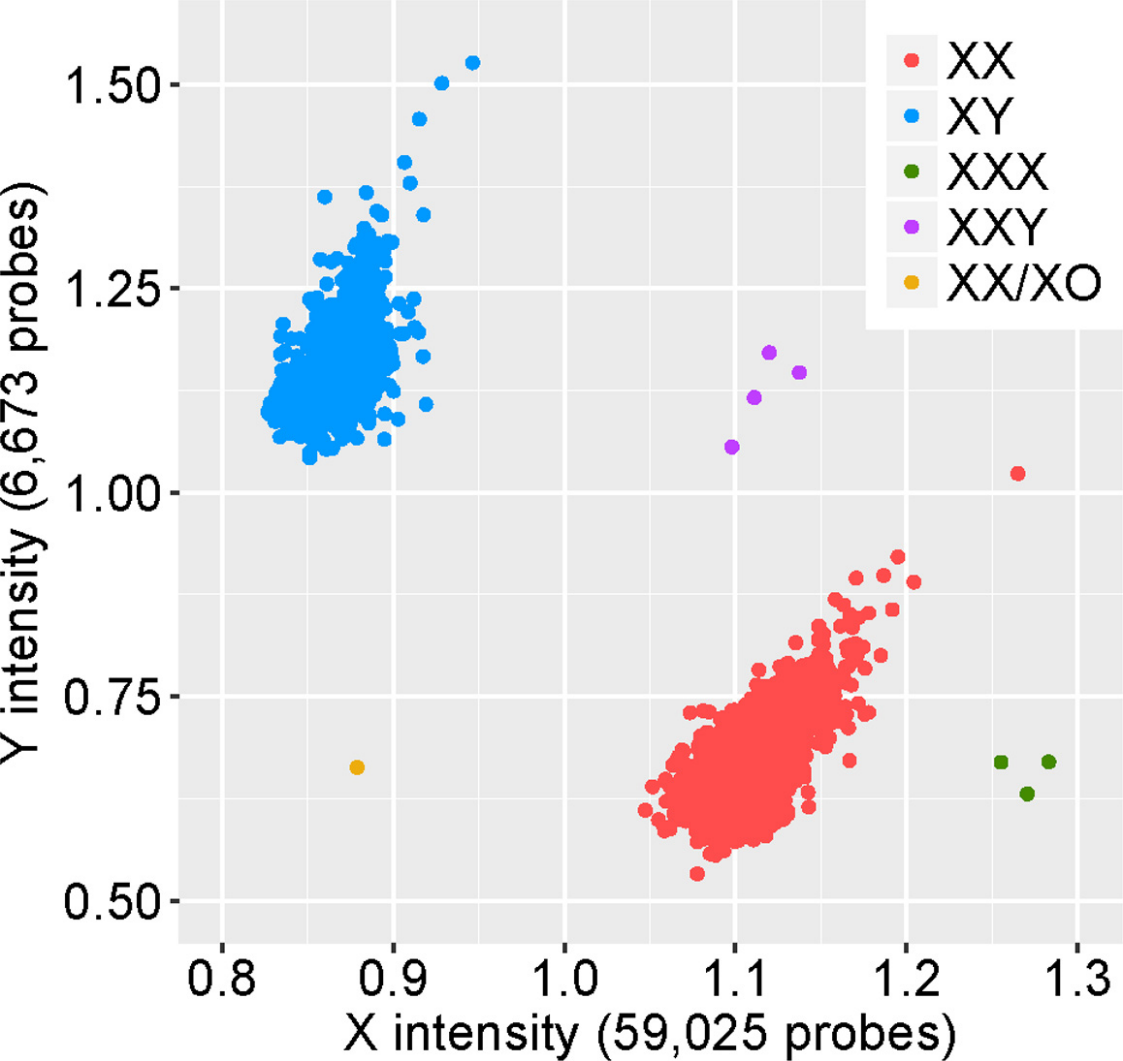
The X and Y chromosome intensity plots showing the cluster of males (blue) and female genotypes (red). Individuals with sex chromosome aneuploidies are shown as indicated in the figure legend. Sample sizes are given in the axis labels

**Figure 2:**
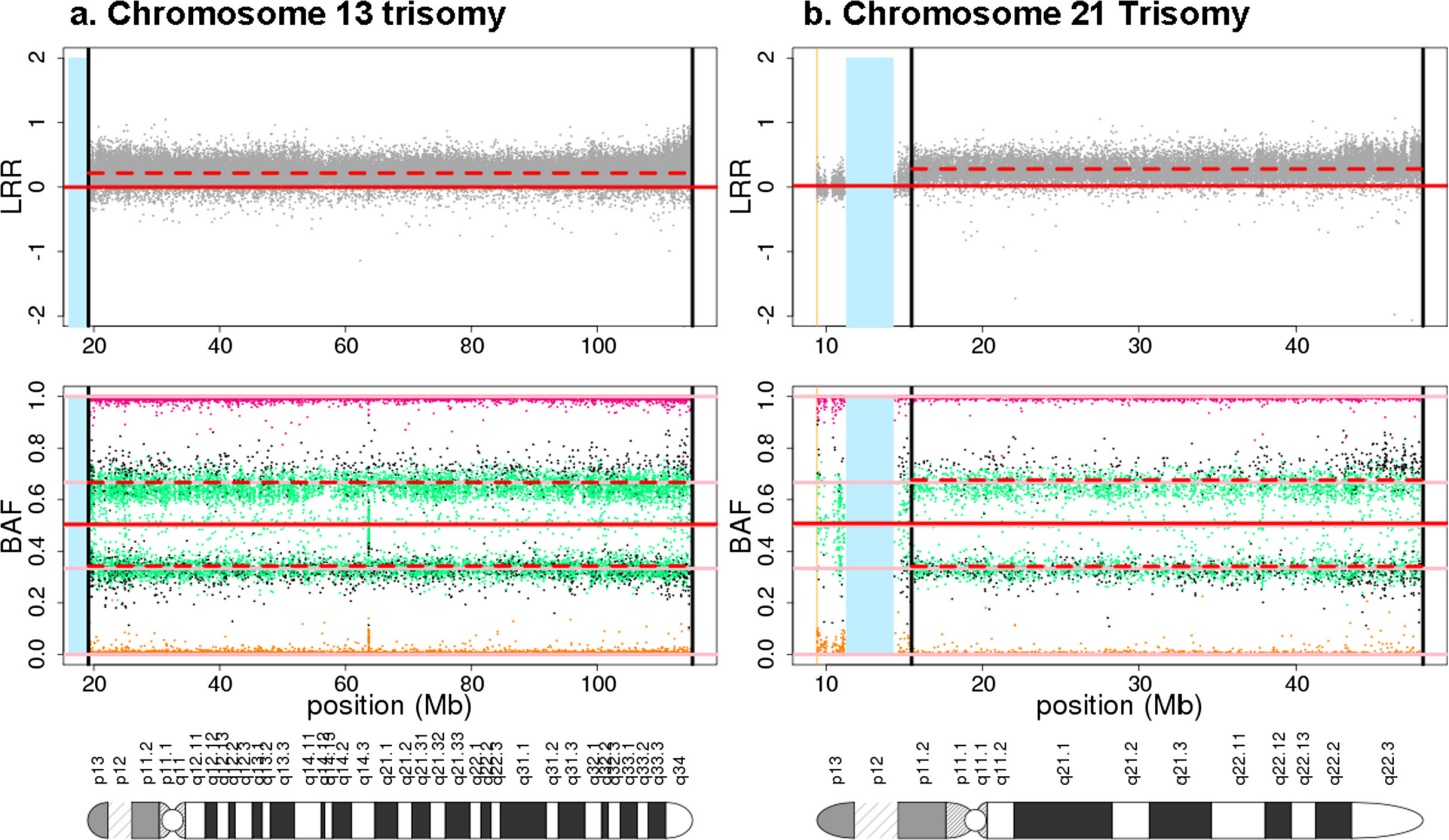
Representaive examples of trisomy 13(A) and 21(B) detected in this study. A pair of BAF and LRR plots is shown for each autosomal aneuploidy. Each plotted point is a single SNP and BAF plots are color‐coded by genotype call (orange = AA, green = AB, fuchsia = BB, black = missing). The vertical black lines indicate the breakpoint(s) of the anomaly. The vertical blue rectangle is the centromeric gap. Horizontal pink lines are drawn at 0, 1/3, 1/2, 2/3, and 1 in the BAF plots. The solid horizontal red line in each plot is the median value for nonanomalous regions of the autosomes. The horizontal dashed red line is the median value within the anomaly. The elevated LRR and the split in the BAF heterozygous band at 1/3 and 2/3 indicate trisomic genotypes: AAA (BAF = 0), AAB (BAF = 1/3), ABB (BAF = 2/3), and BBB (BAF = 1). A typical state of diploid alleles shows three BAF bands: AA (BAF = 0), AB or BA (BAF = 0.5), BB (BAF = 1) and LRR centered at 0

The clinical information of all individuals with sex aneuploidies, trisomies, chromosomal anomalies, large deletions and duplications are described in Tables 1, 2, 3, respectively. We identified three individuals with XXX, four individuals with XXY and one with mosaic XX/X (see Figure 1). They could be mosaics and not actually have the syndromic phenotypes generally associated with these sex chromosome anomalies. We also identified three individuals with Trisomy 13 (Patau syndrome), and 10 individuals with Trisomy 21.Representative BAF and LRR plots from this study for each of these aneuploidies are shown in Figure 2.

**Table 1:** Individuals with sex aneuploidies

Observed clinical sex	Sex annotation from genotype analysis	Cleft type	Cleft description	Additional clinical feature
F	XX/X	CLP	Unilateral – right	
M	XXY	CL	Unilateral – left	Simonart bands
F^a^	XXX	Case mom		
M	XXY	CPO	Submucous cleft palate	
M	XXY	Control		
M	XXY	Control		
F	XXX	CPO	Soft palate	VPI

Note

CL, cleft lip; CLP, cleft lip and palate; CPO, cleft palate only; F, females; M, males; VPI, Velo‐pharyngeal Insufficiency.

a Unaffected case mom.

**Table 2:** Individuals with trisomy, age at recruitment and maternal age at child's delivery

Trisomy	Cleft type/Control	Proband sex	Age at recruitment	Mother age when proband was born	Father age when proband was born	Additional clinical feature
Trisomy 13	CLP	F	3 weeks	45	50	Right unilateral microphthalmia. Hexadactyly of both hands (fingers). Hexadactyly of left foot (toes)^a^
Trisomy 13	CLP	F	Unknown			
Trisomy 13	Unknown cleft type	F	Unknown			
Trisomy 21	CLP	M	1 week	30	36	
Trisomy 21	CLP	M	5 months	32.5	46.5	
Trisomy 21	Control	M	10 years	36		
Trisomy 21	Control	M	10 months	43		
Trisomy 21	Control	M	7 months	35		
Trisomy 21	Control	M	15 months	40		
Trisomy 21	Control	M	14 months	34		
Trisomy 21	Control	F	8 years	30	42	
Trisomy 21	Control	F	13 years	24	27	
Trisomy 21	Control	M	10 months	35	40	

Note

a The additional clinical features indicate that this is a syndromic case which was confirmed during our study.

**Table 3:** Individuals with deletions or duplications and clinical description of cleft types

Chromosome	Sex	Case/Control	Cleft type
18	F	Case	CLP
13	F	Case	Unknown cleft type
7	M	Case	CLP
8	M	Case	CLP
8	M	Case	CL
5	F	Case	CL
13	F	Case	CLP
5	F	Case	CLP
18	M	Case	CPO
21	M	Case	CLP
22	M	Case	CPO
18	M	Case	CLP
6	F	Case	CL
9	M	Case	CLP
15	M	Case	CL
4	F	Case	CPO
7	M	Case	CL
5	F	Case	CL
18	F	Case	CPO
8 & 9	M	Case	CLP
15	F	Case mom of CLP	
3	M	Case	CLP
10	F	Case	CPO
5	F	Case mom of CLP	
6	F	Case	CLP
18	F	Case	CLP
18	M	Case	CLP
8	M	Case	CL
7	M	Control	

Note

CL, cleft lip; CLP, cleft lip and palate; CPO, cleft palate only; F, females; M, males.

**Figure 3:**
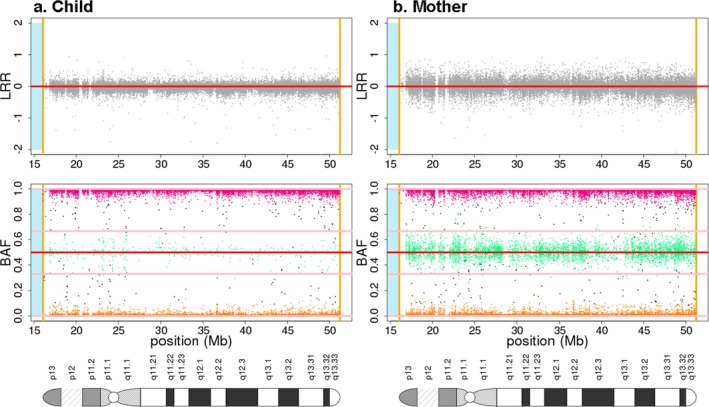
Pair of BAF and LRR plots from chromosome 22 of the individual with paternal UPD (a) and its mother (b). See Figure 2 legend for color‐coding. The pattern of LRR centered at 0 and absence of BAF heterozygous band indicate uniparental disomy. The father was not genotyped but IBS0 estimates between the child and mother on this chromosome are much higher than expected (see results section for details), indicating paternal uniparental disomy

We also identified an apparent case of paternal uniparental disomy (patUPD) on chromosome 22. The UPD is apparent from the nearly complete homozygosity on chromosome 22 (Figure 3) for an affected offspring whose mother was also genotyped. The father was not genotyped. The inference of paternal UPD origin was based on lack of identity by state (IBS0) estimates by chromosome for the mother–offspring pair, for which the expected value is zero. The IBS0 estimate for the mother–offspring pair in Figure 3 using chromosome 22 SNPs is much higher (0.34) than zero and much higher than the IBS0 on any other autosome (IBS0 range 0–0.002) in this pair, or any other parent–offspring pair in this study (IBS0 range 0.001 to 0.0001).

**Figure 4:**
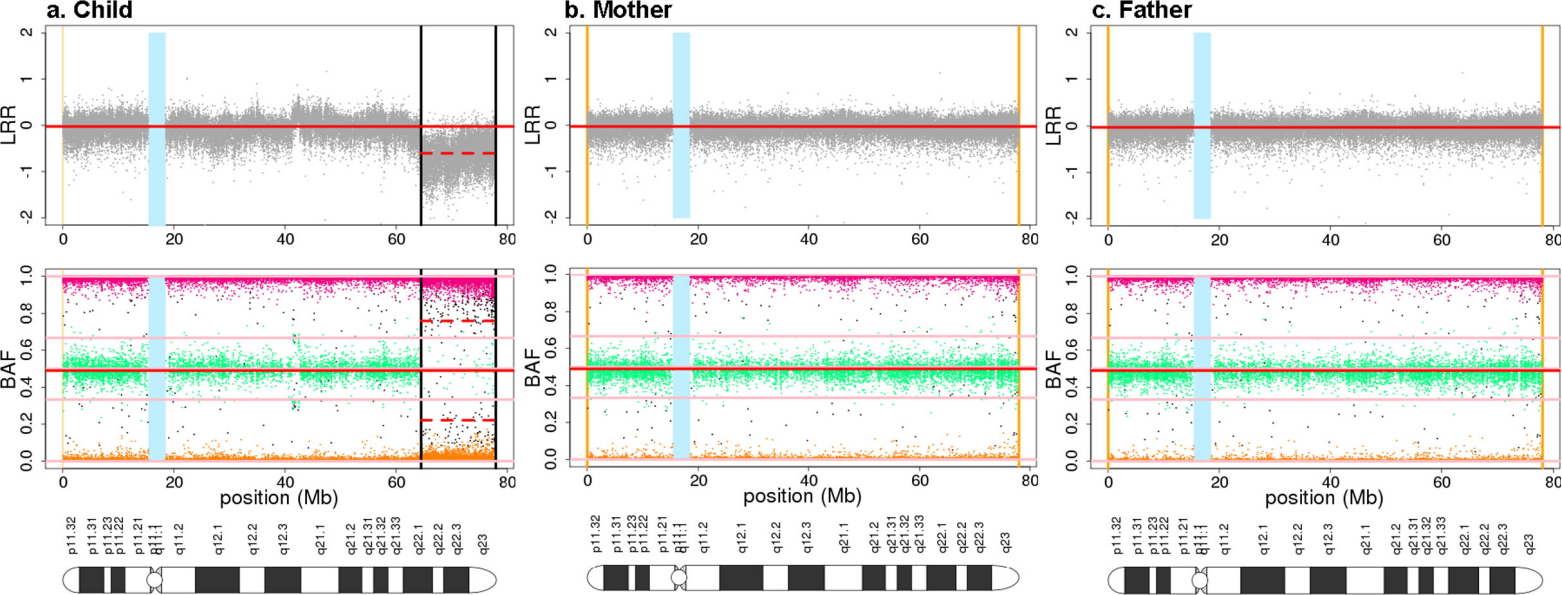
Pair of BAF and LRR plots from chromosome 18 of the affected individual with *de novo* 23MB deletion (a) and its parents(b = mother, c = father). See Figure 2 legend for color coding. In the region of anomaly, the pattern of decreased LRR and absence of the middle heterozygous band indicates deletion. Since the deletion is absent in both parents it is possible that the affected child has a large *de novo* deletion

Furthermore, an affected individual with a *de novo* 23MB deletion on Chromosome 18 was also identified. BAF and LRR plots of this individual and its parents are shown in Figure 4. Other cases with large deletions that are 5 Mbs or more, as well as large duplications were identified in chromosome10, 18, and 21. Each of these was in a mother—affected child pair where mother does not have the deletion. The absence of paternal samples means we could not determine if the deletion is *de novo*. About 30 individuals with large autosomal deletions and duplications were identified (data not provided).

## 4 DISCUSSION

We conducted genotyping of individuals from the African population using the multiethnic genotyping array (MEGA) v2 15070954 A2 to identify genetic variation associated with presumed nonsyndromic clefts. The data cleaning of this dataset allowed us to check the annotated versus genetic sex, confirm identify by descent and identify large chromosomal anomalies. We identified an individual with UPD on chromosome 22 and the result of our analysis strongly indicates that the UPD observed is of paternal origin. Previous UPD associated with clefts has been reported in other chromosomes and are mainly due to maternal UPDs as a result of advanced maternal age during pregnancy leading to trisomy rescue and error (Romanelli et al., 2011). A recent study reported an association with maternal UPD (matUPD) on chromosome 21 with Bartsocas Papas Syndrome (Leslie et al., 2015). In the absence of any other obvious large genetic aberration, it is possible that the affected individual has a recessive form of clefting arising from a heterozygous father and unmasked by the patUPD on chromosome 22. Furthermore, we cannot rule out the possibility that the observed anomaly might be an incidental occurrence and other genomic events such as SNPs or small chromosomal anomalies may be causing the phenotype either in combination with this chromosomal anomaly or independently. In addition, this could well be a mosaic anomaly and, if so, it might not occur in the tissues that lead to cleft palate.

The *de novo* deletion in chr18 is particularly interesting. It is likely due to gametogenesis because both parents do not carry the deletion and we suspect it may account for clefting since the parents are clinically normal. The deleted region overlaps a 23MB region found deleted in individuals with a spectrum of developmental anomalies including clefts as reported in DatabasE of genomiC varIation and Phenotype in Humans using Ensembl Resources (DECIPHER (https://decipher.sanger.ac.uk/index)). The 23MB *de novo* deletion included the *ZADH2* gene which has been reported in DECIPHER for most individuals with developmental disorders including clefts. Over 50% of individuals with large deletions including the child with a *de novo* deletion had CLP. Large deletions in individuals with CL/P have been previously reported (Maarse et al., 2012).

There are inconsistent reports of clefting in some individuals with sex aneuploidies (Perrotin et al., 2001). The individuals with these sex aneuploidies have the different types of clefts that can be seen in nonsyndromic cleft cases. Most often, they appear as NSC and thus will require additional genetic diagnosis. Three of the children had trisomy 13 otherwise known as Patau syndrome. These infants do not usually survive beyond the first few days of life (Rasmussen, Nielsen, & Dahl, 1982). We followed up with their mothers and confirmed that the three infants died shortly after birth. This is consistent with the expectation that about 90% of children with this syndromes do not survive beyond the first few days of life (Rasmussen et al., 1982). These children were recruited into this study at birth and may have been incorrectly enrolled as nonsyndromic clefts.

It is very important to carry out detailed clinical evaluation of children with apparent nonsyndromic clefts by individuals with the skill to investigate family history, environmental exposures and do a thorough clinical exam on child and parents. Genetic testing to exclude chromosomal anomalies may be indicated by the findings of these exams and also in cases of prenatal detection of clefting where phenotyping is more difficult. This study is limited by clinical examination information available at birth or within a few days after delivery. Therefore a detailed periodic exam is advised in order to identify associated anomalies that may become clinically obvious days after birth. This is because over 15% of clefts with associated anomalies are caused by chromosomal defects (Rasmussen et al., 1982) and genetic testing is advised in clefts with associated anomalies. Genetic testing may not be readily available in resource‐limited setting but clinicians should share any identified genetic findings and place them in context for the families during routine clinical visits and evaluations.

## CONFLICTS OF INTEREST

None to declare.
